# Is *miR-29* an oncogene or tumor suppressor in CLL?

**DOI:** 10.18632/oncotarget.129

**Published:** 2010-07-16

**Authors:** Yuri Pekarsky, Carlo M. Croce

**Affiliations:** Human Cancer Genetics Program and Department of Molecular Virology, Immunology and Medical Genetics, OSU School of Medicine, Ohio State University, Columbus, Ohio

## Abstract

B-cell chronic lymphocytic leukemia (CLL) is the most common leukemia in the Western world. CLL occurs in two forms, aggressive and indolent. Aggressive CLL is characterized by high ZAP-70 expression and unmutated IgH V_H_; indolent CLL shows low ZAP-70 expression and mutated IgH V_H_. We recently found that *miR-29* is upregulated in indolent human B-CLL, compared to aggressive B-CLL and normal CD19+ B-cells. To determine the role of *miR-29* in CLL, we generated transgenic mice overexpressing *miR-29* in mouse B-cells. Recently we reported that *miR-29* transgenic mice develop indolent CLL phenotype. Interestingly, our previous findings suggest that *miR-29* targets expression of *TCL1*, a critical oncogene in aggressive CLL, indicating that *miR-29* might function as a tumor suppressor in CLL. Here we discuss these results and provide additional insights into function of *miR-29* in CLL.

Chronic lymphocytic leukemia (CLL) is the most common human leukemia, accounting for ~10,000 new cases diagnosed each year in the United States (~30% of all leukemia cases) [1]. CLL is mostly a disease of elderly people, with the incidence increasing linearly with each decade [1,2]. This disease occurs in two forms, aggressive and indolent, both forms are is characterized by the clonal expansion of CD5 positive B-cells [1,2]. Aggressive CLL is characterized by high ZAP-70 expression and unmutated IgH V_H_; indolent CLL shows low ZAP-70 expression and mutated IgH V_H_ [1,2].

MicroRNAs are endogenous non-coding RNAs 19-25 nucleotides in size [3]. Recent studies have shown that microRNAs play important roles in various cellular processes including DNA methylation [4], cellular growth, differentiation and apoptosis [5]. Recent studies revealed that nearly half of human microRNAs are located within fragile sites and genomic regions altered in various cancers [6]. Numerous reports demonstrated that, as protein coding genes, microRNAs differentially express in a number of cancers, indicating that individual microRNAs could play tumor suppressor or oncogenenic roles in cancer pathogenesis [7].

Several recent studies demonstrated that microRNA expression profiles can be used to distinguish normal B-cells from malignant CLL cells and that microRNA signatures are associated with prognosis and progression of CLL [6,8]. Specifically, a signature profile was reported, describing 13 microRNAs that differentiate aggressive and indolent CLL [6].

Tcl1 is a critical molecule in the pathogenesis of CLL [9]. Mouse model studies conclusively demonstrated that deregulation of *TCL1* is initiating event in the development of the aggressive form of CLL [10,11], in fact recent studies showed that Tcl1- driven mouse CLL closely resembles the aggressive form of human B-CLL and the analysis for V_H_ mutations showed that all the CLLs in transgenic mice carried unmutated V_H_ genes in accordance with the aggressive phenotype [12]. We, and others, reported that the aggressive form of human B-CLL shows the highest *TCL1* expression levels [13,14]. Several years ago we investigated whether microRNAs regulate *TCL1* expression in CLL. We demonstrated that *miR-29* and *miR-181* target *TCL1* expression in CLL [14]. Interestingly, of the four down-regulated microRNAs in aggressive CLL versus indolent B-CLL, three are different isoforms of *miR-29* (*miR-29a-2, miR-29b-2* and *miR-29c*) [6], strongly suggesting that *miR-29* and *TCL1* interactions play an important role in the pathogenesis of aggressive CLL [14]. The fact that *miR-29* targets expression of *TCL1*, a critical oncogene in aggressive CLL, indicates that *miR-29* might function as a tumor suppressor in CLL.

As noted above, we have previously reported that *miR-29* expression is down-regulated in aggressive *versus* indolent CLL [8,14], but these reports did not examine *miR-29* expression in CLL *versus* normal CD19+ B-cells. In our latest publication in PNAS we examined expression of *miR-29a* and *miR-29b* in 29 aggressive CLL samples, 33 indolent CLL samples and two normal CD19+ B-cell controls [15]. We found that *miR-29a* and *miR-29b* expression was 4-4.5 fold higher in indolent CLL, when compared with normal CD19+ B-cells [15]. Table 1 shows summary of *miR-29* expression in CLL from three studies. Deletion of chromosome 11 in CLL usually indicates most aggressive phenotype. Interestingly, CLL samples showing this particular deletion express lowest levels of *miR-29*. These data clearly indicate that *miR-29a* and *miR-29b* expression is clearly down-regulated in aggressive CLL *versus* indolent CLL.

**Table 1:** *Mir-29* expression in CLL

Study ref.	Number of samples	Method	Results
[8]	Aggressive CLL 36 Indolent CLL 47	Microchip	*miR-29a* and *miR-29b* down-regulated in aggressive CLL vs.indolent CLL
[14]	Aggressive CLL 25 Aggr. CLL (Del. Chr. 11) 32 Indolent CLL 23	Microchip	*miR-29a* and *miR-29b* down-regulated in aggressive CLL (Del. Chr 11) vs.indolent CLL (~2 fold) *miR-29b* down-regulated in aggressive CLL vs. indolent CLL (~2 fold)
[15]	Aggressive CLL 29 Indolent CLL 33 Normal cord blood B-cells 2	Real Time RT-PCR	*miR-29a* down-regulated in aggressive CLL vs. indolent CLL (~1.5 fold) *miR-29a* and *miR-29b* up-regulated in aggressive CLL vs. normal B-cells (~3 fold) *miR-29a* and *miR-29b* up-regulated in indolent CLL vs. normal B-cells (~4-5 fold)

Although deregulation of a specific gene in a certain type of cancer suggests a potential involvement in the malignancy, the final proof of the involvement of this gene in the pathogenesis of this disease requires generation of animal models showing the same malignant phenotype. To elucidate the role of *miR-29* in B-cell leukemias we generated transgenic mice overexpressing *miR-29* in B-cells. Very recently we reported the phenotype of this mouse model [15].

Immunophenotypic profile of spleen lymphocytes from *miR-29* transgenics showed increased populations of CD5+CD19+IgM+ B-cells, a characteristic of CLL. At the age of 12-24 months markedly expanded CD5^+^ B-cell population was evident in spleens of 34 of 40 (85%) *miR-29* transgenic mice; ~50% of B-cells in these transgenics were CD5 positive [15]. Interestingly, of 20 *miR-29* transgenic mice followed to 24-26 months of age, only 4 (20%) developed frank leukemia and died of the disease. Because almost all *miR-29* transgenics showed expanded CD5+CD19+IgM+ Bcell populations, but only 20% develop frank leukemia we concluded that *miR-29* transgenics develop a disease similar to indolent CLL [15]. In addition, *miR-29* mice showed significant increases in % of leukemic cells with age. In mice younger than 15 months, CD5+ leukemic cells represented only ~20% of total B-cells; in contrast, at the age of 20-26 months, on average, >65% of all B-cells were CD5+. These results show gradual progression of indolent B-CLL in *miR-29* transgenics [15]. To determine whether leukemic cells from *miR-29* mice divide, we measured the proliferative capacity of CD5+ leukemic B-cells in comparison with WT CD19+ splenic lymphocytes. BrdU incorporation experiments showed significant proliferation in *miR-29* transgenic B-cells, while no proliferation was detected for CD19+ WT lymphocytes [15]. These data suggest that *miR-29* over-expression plays an important role in promoting B-cell proliferation. Since progressive hypogammaglobulinemia and immune incompetence are important features of human CLL [16], we analyzed serum levels of immunoglobulins and the immune response to SRBC antigen in the serum of *miR-29* transgenics and wild type siblings. We found that in *miR-29* transgenics immune response to SRBC antigen and serum levels of immunoglobulins were significantly decreased [15]. These data confirmed our initial observations that *miR-29* transgenic mice develop disease that mimics indolent human CLL.

Previously we reported that *miR-29* is one of two microRNAs targeting *TCL1*, a very important oncogene involved in the pathogenesis of aggressive CLL [14]. We also showed that *TCL1* and *miR-29* expression levels are inversely correlated in CLL, and that *miR-29* expression is down-regulated in aggressive CLL *versus* indolent CLL [14]. These data suggest possible tumor suppressor function for *miR-29* in CLL. Very recently we reported that *miR-29* is up-regulated in indolent CLL compared to normal B-cells [15]. We also found that *miR-29* transgenic mice showed expanded CD5+CD19+IgM+ B-cell populations and develop a disease very similar to indolent human CLL [15]. This implies that up-regulation of *miR-29* initiates or at least significantly contributes to the pathogenesis of indolent CLL and can function as an oncogene. How can we reconcile these differences? Figure 1 shows our current view of the function of *miR-29* in CLL. Since *TCL1* is mostly not expressed in indolent CLL [14], it likely does not play an important role in indolent CLL. Thus its down-regulation due to *miR-29* overexpression does not slow indolent CLL development. We believe that up-regulation of *miR-29* expression is not sufficient to cause aggressive CLL. In contrast, up-regulation of Tcl1 is absolutely required for the initiation of the aggressive form of CLL. In the other hand, down-regulation of *miR-29* expression in aggressive CLL (compared to the indolent form) contributes to up-regulation of Tcl1 and development of aggressive CLL.

**Fig. 1:**
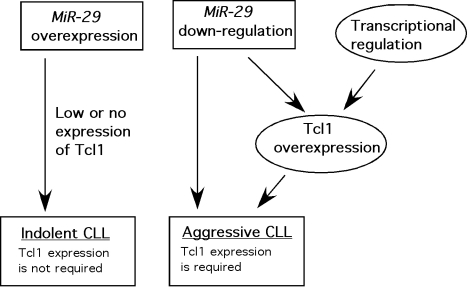
Role of *miR-29* in CLL.

Since in all human B-CLL samples *miR-29a* expression levels were >20-fold higher compared to *miR-29b* [15] and both microRNAs share the same seed sequence, it is likely that most of effects of expression of *miR-29ab* cluster in CLL can be attributed to *miR-29a*. *MiR-29* functions as an oncogene in B-cells by regulating its targets. Targetscan software predicts over 800 targets for *miR-29*. This list contains multiple oncogenes and tumor suppressor genes. Previous reports showed that *miR-29* can target *TCL1*, *MCL1* and *CDK6* oncogenes [14,17,18]; we propose that *miR-29* targets possible tumor suppressor peroxidasin [15]. Clearly, interactions of *miR-29* with these genes contribute to its function, but it is very likely that hundreds of unknown interactions of *miR-29* with its targets hold keys to its oncogenic role in CLL.

MicroRNA expression profiles were extensively studied in a number of hematological malignancies as well as in solid tumors [7]. These studies resulted in identification of several microRNAs that might function as tumor suppressors or oncogenes [7]. However, there have been only two reports demonstrating that dysregulation of a single microRNA (or a cluster) can cause cancer. The first such study defined an initiation role of *miR-155* in B-cell acute leukemias [19]. Another study demonstrated that knockout of *miR-15/16* led to the development of indolent B-cell malignancies [20,21]. In both cases previously published reports identified *miR-155* as an oncogene and *miR-15/16* as a tumor suppressor. In contrast, there has not been a clear consensus regarding the function of *miR-29* in this respect. Previous studies showed that *miR-29* expression was down-regulated and correlated with poor prognosis in mantle cell lymphoma [22], its re-expression caused apoptosis AML cells [17] and suppressed tumorigenicity in lung cancer cells [23]. On the other hand, in addition to our report [15], dysregulation of *miR-29* expression in myeloid cells was found to cause AML in a mouse model [24], and its overexpression was reported in metastatic breast cancer [25]. Thus, it is apparent that depending on cellular contexts *miR-29* can function as an oncogene or a tumor suppressor.
